# Drenched Silicon Suppresses Disease and Insect Pests in Coffee Plant Grown in Controlled Environment by Improving Physiology and Upregulating Defense Genes

**DOI:** 10.3390/ijms23073543

**Published:** 2022-03-24

**Authors:** Jingli Yang, Jinnan Song, Byoung Ryong Jeong

**Affiliations:** 1 Department of Horticulture, Division of Applied Life Science (BK21 Four), Graduate School of Gyeongsang National University, Jinju 52828, Korea; yangmiaomiaode@gmail.com (J.Y.); jinnansong93@gmail.com (J.S.); 2 Institute of Agriculture and Life Science, Gyeongsang National University, Jinju 52828, Korea; 3 Research Institute of Life Science, Gyeongsang National University, Jinju 52828, Korea

**Keywords:** silicon, antioxidants, biotic stress, growth improvement, induced resistance, microscopy, photosynthesis, physiology

## Abstract

Plant disease and insect pests are major limiting factors that reduce crop production worldwide. The ornamental indoor cultivation cash crop dwarf coffee *Punica arabica* ‘Pacas’ is also troubled by these issues. Silicon (Si) is one of the most abundant elements in the lithosphere and positively impacts plant health by effectively mitigating biotic and abiotic stresses. Several studies have shown that Si activates plant defense systems, although the specific nature of the involvement of Si in biochemical processes that lead to resistance is unclear. In our study, Si significantly promoted the growth and development of dwarf coffee seedlings grown in plant growth chambers. More than that, through natural infection, Si suppressed disease and insect pests by improving physiology (e.g., the strong development of the internal structures of roots, stems, and leaves; higher photosynthetic efficiency; more abundant organic matter accumulation; the promotion of root activity; the efficient absorption and transfer of mineral elements; and various activated enzymes) and up-regulating defense genes (*CaERFTF11* and *CaERF13*). Overall, in agriculture, Si may potentially contribute to global food security and safety by assisting in the creation of enhanced crop types with optimal production as well by mitigating plant disease and insect pests. In this sense, Si is a sustainable alternative in agricultural production.

## 1. Introduction

Plant disease and insect pests are the most significant constraints on agricultural productivity across the world. Almost all crops are susceptible to plant pathogens and insect pest damage. Annual crop losses due to illness is estimated to be over 25% [1], and 10.8% of the total worldwide production is lost due to pests [2]. Such losses can have a significant financial and economic impact on the local economy, perhaps leading to famine. Thus, the timely control of plant diseases and insect pests must be prioritized.

**Figure 1:**
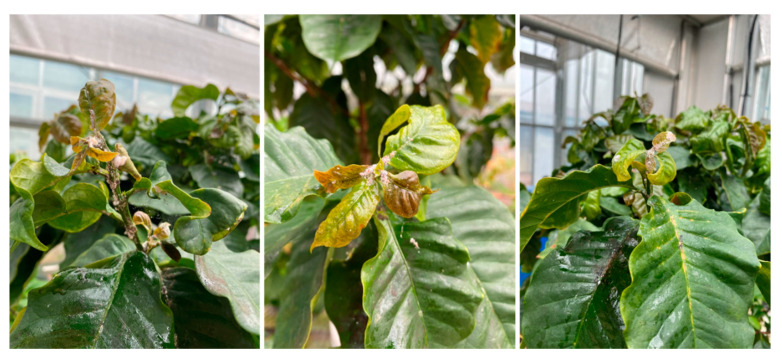
Disease and insects in the coffee plants grown indoors.

The dwarf coffee *Punica arabica* ‘Pacas’ is an attractive little specimen with glossy green leaves and a compact growth habit. It makes a surprisingly good potted indoor plant. Native to Central America, it is a natural mutation of Bourbon. Similar to other widely cultivated Bourbon mutants, it has a single-gene mutation that causes the plants to grow shorter (dwarfism) with the chief virtue: the plant’s small size leads to higher potential yields and the possibility of placing plants closer together to increase total fruit production on a farm. However, it is highly susceptible to major diseases and insects (https://varieties.worldcoffeeresearch.org/varieties/pacas) (accessed on 15 February 2022). Coffee plants grown indoors will sometimes suffer from infestations of mealybugs, aphids, and mites [3]. Signs of infestation include tiny webs, clumps of white powdery residue, or visible insects on the plant (Figure 1), and this has become the main problem in indoor coffee cultivation. Traditional management strategies involve the use of pesticides and cultural practices that include the application of soil amendments for enhancing the resistance of the host plant [4]. This may include the utilization of various fertilizers (NPK) and elements such as zinc (Zn), boron (B), magnesium (Mg), and silicon (Si). However, insecticides are potentially harmful to human health, especially in relatively closed indoor conditions.

Initially, Si was thought to be a non-essential component for plant development and sustenance [5]. However, the current study has discovered that it is a non-toxic, useful, and abundant element that participates in a wide range of plant activities [6,7,8]. Taking plant physiology as an example, Si can reinforce photosynthesis at the cost of lower transpiration, thus benefiting nitrogen metabolism and carbon accumulation [9,10,11]. Si is absorbed and forms a double silicate layer on the leaf epidermis, allowing better leaf architecture and greater light assimilate capacity [12]. Additionally, Si uptake and transport in the lateral roots are accomplished via the Si accumulators, but in shoots, they are regulated by the transpiration rate [13]. One of the most beneficial roles of Si that has been demonstrated is that it helps plants manage biotic and abiotic stresses [14]. Moreover, many studies have suggested that Si plays an important role in increasing the rigidity of the cell walls of plants, leading to a more erect plant [15]. In commercially significant crops, such as tobacco, cotton, and sorghum, Si promotes plant resistance to bacterial, fungal, and viral diseases, as well as insect infestations [16,17,18,19]. In addition, researchers have found that Si can stimulate the plant defense system against the infection of *P. ultimum*, which makes plants prone to damping off [20]. Equally as important, Si has a great potential in mitigating the symptoms caused by *Fusarium* wilt, which is believed to destroy water translocation, inducing drooping foliage, leaf chlorosis, and other wilt symptoms [21].

**Figure 2:**
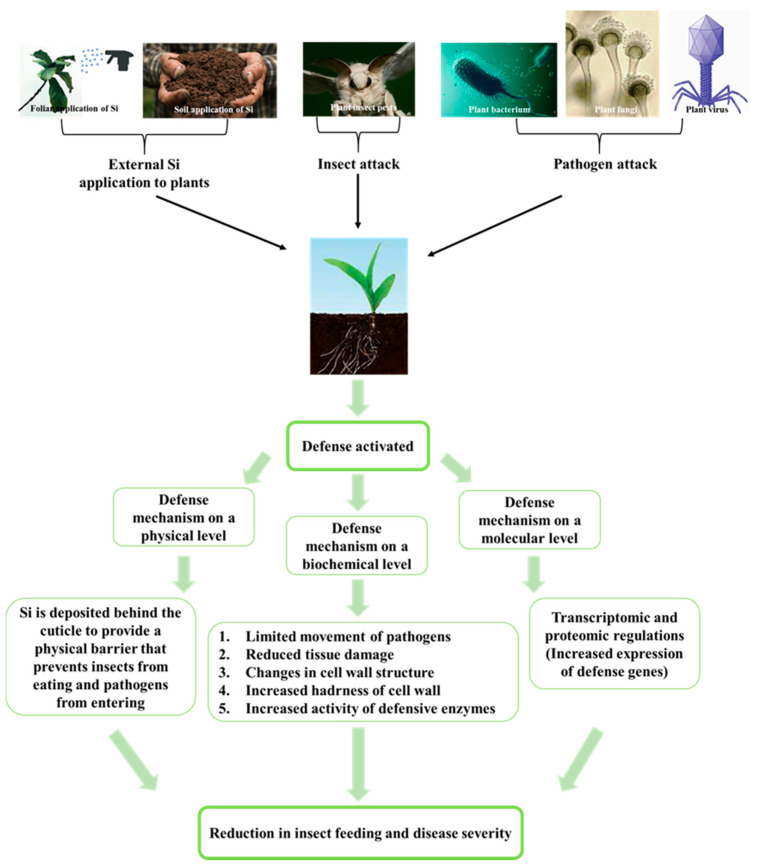
Silicon mediated plant defense against disease and insect pests.

Briefly, in response to insect pests and pathogen attacks on plants, various defense mechanisms are activated or induced by the supplementation of Si to the plants or soil, involving physical, biochemical, and molecular defenses (Figure 2) [22]. The physical mechanism embeds the deposition of Si below the cuticle, which hinders the entry of pathogens and the feeding activity of insect pests. The biochemical defense may involve the stimulation of defense-related enzymes that contribute to reduced tissue damage via herbivory and pathogen movement inside the plant tissues. The molecular defense modulates transcriptomic and proteomic regulations (e.g., improves the expression of defense genes) that improve a plant’s resistance against insect pests and disease.

Numerous studies have confirmed the functions of spraying Si on crop yield; however, researchers have rarely reported the influences of drenched Si on morphophysiological performance, the internal structures of nutritive organs, especially the prevention of disease and pests in indoor cultivated dwarf coffee plants. Our study highlighted the enhancement of drenched Si on suppressing disease and insects by improving physiological characteristics and upregulating defense genes in dwarf coffee *Punica arabica* ‘Pacas’ grown indoors. Moreover, the external Si treatment significantly suppressed indoor coffee plant pests and disease, gradually eliminating the need for pesticides, with this treatment being more environmentally friendly and avoiding potential health hazards. It also provides more development space for this ornamental indoor cultivation plant.

## 2. Results

### 2.1. Morphology and Plant Growth Parameters

**Figure 3:**
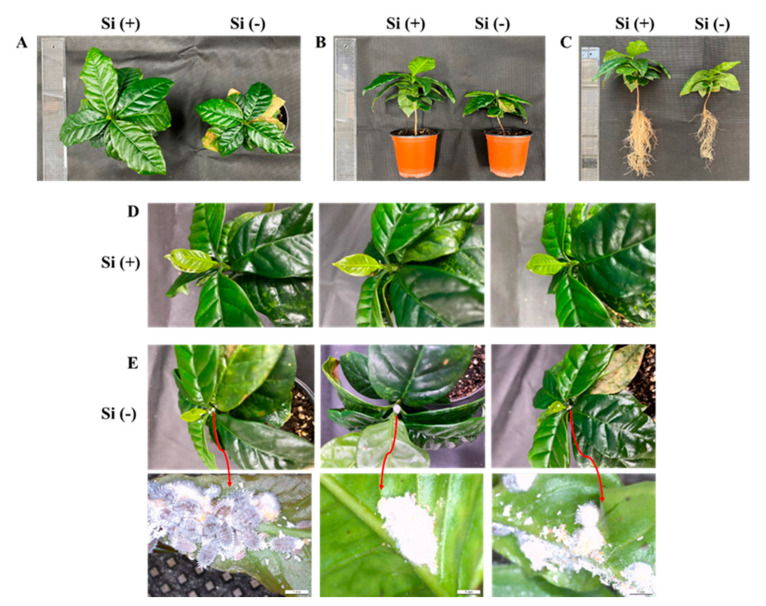
Effect of 30 days silicon application on morphology (**A**–**C**), disease, and insects (**D**,**E**) in the coffee plants grown in plant growth chambers. Si (+) and Si (−) refer to 75 and 0 mg·L^−1^, respectively. The red arrows indicate high magnification microscope photographs of plant disease or insects. Bars indicate 1 mm.

The coffee seedlings were cultivated with or without drenched Si in plant growth chambers for 30 days until disease or insects could first be observed (through natural infection). Morphology and plant growth parameters responded significantly to Si application. In the present experiments, significant increases in plant height, canopy diameter, stem diameter, shoot fresh and dry weight, and leaf number and size were observed under “multipurpose nutrient solution (MNS) + Si” treatment (experimental group) when compared with only the application of the MNS (control group) (Figure 3A,B and Table 1). Furthermore, Si induced upright leaves and significantly reduced the adaxial angle of the top^1st^ leaves, thereby improving the canopy in terms of light penetration and increasing the optimal leaf area (Table 1). Moreover, the lower leaves of the Si treated plants were a darker green, thus better disposed for light interception, were noticeably rougher, and, especially, had thicker stems, in comparison with these features of the control plants. In addition, the leaves of the Si (−) plants were prone to wilt and senesced earlier than those of the Si (+) plants. Remarkably, the root length, root amount, root fresh weight, and root dry weight in plants treated with Si were greater than those of the control (Figure 3C and Table 1). Moreover, Si performed important functions in the resistance against disease or insects. After 30 days of natural infection cultivation, some diseases appeared in the leaves of the control plants, which, turning increasingly yellow, became withered. At the same time, a kind of plant white filamentous fungi and white mealybugs (*Family Pseudococcidae*) were also observed in the control plants (Figure 3E). However, the experimental plants were well developed without any disease speckles, pathogens, or insects (Figure 3D). Overall, the comprehensive data (Figure 3 and Table 1) showed that Si not only promoted plant morphological development but also played an important role in disease or insect mitigation and resistance.

### 2.2. Anatomical Features of Leaves, Stems, and Roots

**Figure 4:**
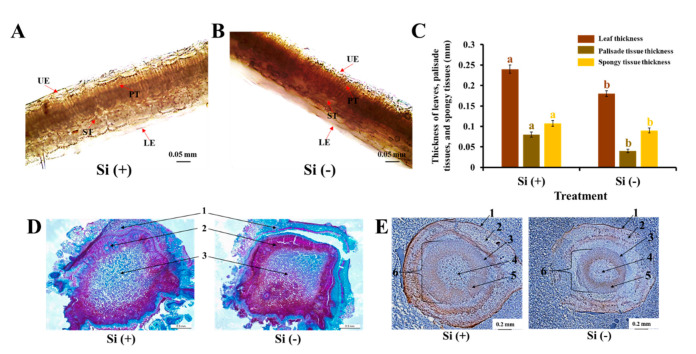
Effect of 30 days of silicon application on anatomical features of leaves, stems, and roots in the coffee plants grown in plant growth chambers. Leaf structure (**A**,**B**) and column diagram of leaf structures (**C**); UE, upper epidermis; LE, lower epidermis; PT, palisade tissues; ST, spongy tissues; bars indicate 0.05 mm. Stem structure (**D**); 1, 2, and 3 refer to the cortex, vascular bundle, and pith, respectively; bars indicate 0.3 mm. Root structure (**E**); 1, 2, 3, 4, 5, and 6 refer to the epidermis, cortex, pericycle, xylem, phloem, and stele, respectively; bars indicate 0.2 mm. Si (+) and Si (−) refer to 75 and 0 mg·L^−1^, respectively. Vertical bars indicate the means ± standard error (*n* = 6). Different lowercase letters indicate the significant separation within treatments by the Duncan’s multiple range test at *p* ≤ 0.05.

Moreover, we further studied the application of Si on the improvement of the anatomical features of leaves, stems, and roots at the cellular level. A transmission electron microscope was used to observe the effect of Si on the ultrastructure of coffee leaves, stalk pith tissues, and roots. The leaf thickness was greater in the Si-treated plants compared to those without Si. Other indices of leaf structure performance were also higher in the Si (+) plants. Si induced a well-developed and thick epidermis, and neatly arranged epidermal cells could be observed on both the upper and lower surfaces of leaves in the Si-treated plants (Figure 4A). Interestingly, the leaves of the experimental plants displayed perfectly developed palisade and spongy tissues with more compact structures when compared with the Si (−) plants. Additionally, Si markedly promoted leaf thickness (Figure 4A–C). In Si (−) treatment, the parenchyma cells of the stalk pith had an abnormal structure, and the cell wall between the upper and lower adjacent cells was broken, resulting in the loss of connections between vascular cells and worsened supporting capacity. In addition, the parenchyma cells of the cortex and main pith in the Si (−) plants were loosely arranged and there was a smaller number of cell layers compared to the Si (+) plants (Figure 4D). Moreover, Si significantly enhanced root thickness, and improved Si nutrition. Si also helped to maintain a well-developed root with clear and complete structures, and the tight arrangement of root cells with thick cell walls effectively prevented the invasion of pathogens (Figure 4E). Overall, improved Si nutrition promoted the development of vegetative organs in dwarf coffee plants through maintaining cell structure stability, preventing the expansion of intracellular space, reinforcing cell walls, and forming materials between cells in order to reduce the chances of pathogen invasions. All of which further promoted plant photosynthesis and induced greater physiological parameters.

### 2.3. Morphological Characteristics of Stomata

**Figure 5:**
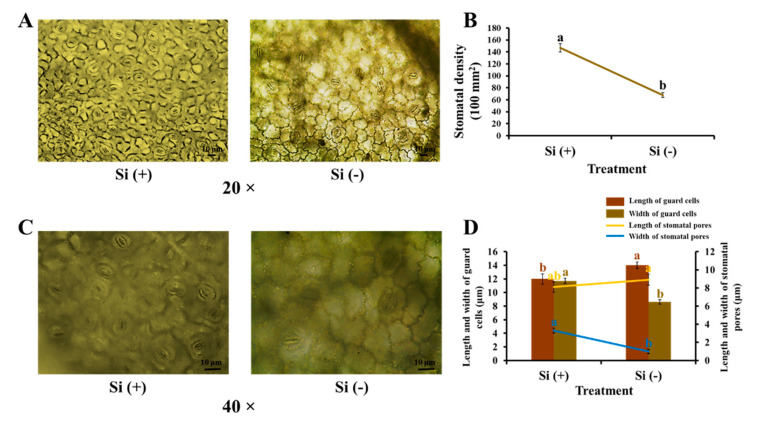
Effect of 30 days of silicon application on stomatal characteristics in the coffee plants grown in plant growth chambers. Stomatal density (**A**,**B**), 20 times amplification. Stomatal morphology (**C**,**D**), 40 times amplification. Si (+) and Si (-) refer to 75 and 0 mg·L^−1^, respectively. Vertical bars indicate the means ± standard error (*n* = 6). Different lowercase letters indicate the significant separation within treatments by the Duncan’s multiple range test at *p* ≤ 0.05. Bars indicate 10 μm.

In the previous inquiry, exogenous Si significantly influenced the leaf and stem structures of coffee plants (Figure 4A–D). Stomata are tiny openings or pores in plant tissue that allow the exchange of gases which are typically found in plant leaves but also in some stems. Therefore, we further explored the effect of Si application on stomatal characteristics. As we know, Si increased leaf thickness, thus, Si (−) samples became more pellucid and made it easier to observe the stomata than in the case of the Si (+) leaves after a series of decolorization processes. Si (+) induced prominent high stomatal density with plump guard cells, markedly open pores, and a short-and-wide shape in the experimental leaves (Figure 5). Well-developed stomata allow for a higher gas change efficiency and assist in transpiration, which helps to promote photosynthesis and resist stress.

### 2.4. Chlorophyll Content, Photosynthesis, and Chlorophyll Fluorescence Characteristics

**Figure 6:**
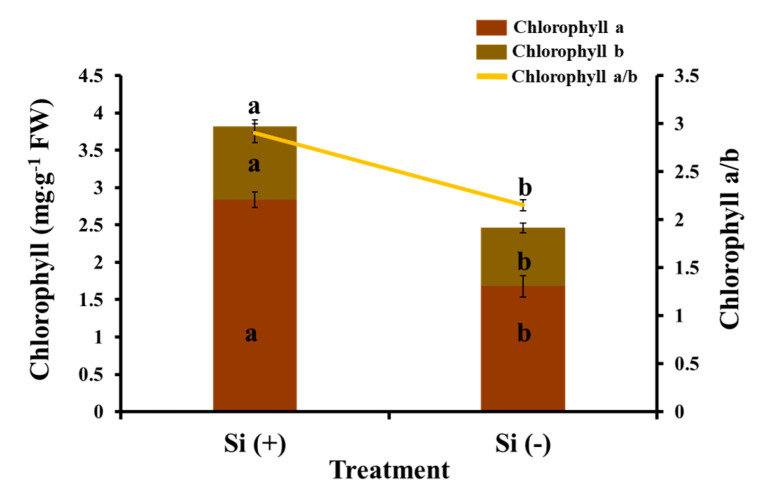
Effect of 30 days of silicon application on chlorophyll content in the coffee plants grown in plant growth chambers. Si (+) and Si (-) refer to 75 and 0 mg·L^−1^, respectively. Vertical bars indicate the means ± standard error (*n* = 6). Different lowercase letters indicate the significant separation within treatments by the Duncan’s multiple range test at *p* ≤ 0.05.

One of the most important factors for photosynthetic activity is the photosynthetic pigment. The contents and ratios of chlorophyll are given in (Figure 6). Applying silicon increased the chlorophyll content in coffee leaves. The results show a dramatic increase in the content of chlorophyll a, chlorophyll b, and chlorophyll a + b in the Si-treated leaves (by about 69.0%, 25.6%, and 55.3%, respectively, in comparison to the control). The ratio of chlorophyll a/b was lowest in the non-Si-treated variant. The results of the photosynthetic parameters show the strong effect of the Si on photosynthetic activity. As shown in Table 2, when compared with the control, Si-application significantly improved the net photosynthetic rate (*P*n), stomatal conductance (*G*s), and intercellular CO_2_ concentration (*C*i), which indicates that silicon application enhanced the transport of gas from the stomata to chloroplasts, maintaining a higher *P*n in leaves, promoting plant growth, and further raising the biomass of the coffee tree. However, a lower transpiration rate (*T*r) was observed in the Si (+) treatment, maybe due to Si deposits between the keratin and epidermal cells of stems and leaves, forming a two-layer structure of keratin and silicon which inhibited transpiration. The other factor which characterizes photosynthetic activity is chlorophyll fluorescence, which provides information about PSII activity in response to environmental factors. Moreover, chlorophyll fluorescence can be considered as an indirect indicator of the state of the integral photosynthetic process due to the functional relationship between PSII and the other components of the photosynthetic apparatus. Chlorophyll fluorescence in dark-adapted leaves (Table 2) varied dramatically between Si (+) and Si (−) treatments. The application of Si to the nutrient solution caused an increase in the maximal PSII quantum yield (*F*v/*F*m), the photochemical efficiency of PSII (*F*v′/*F*m′), non-photochemical quenching (NPQ), and the coefficient of photochemical quenching (*qP*) when compared to the variant treated only with nutrient solution. Overall, Si definitely enhanced PSII activity, the photosynthetic electron transport velocity, and photosynthetic efficiency, which is possibly an important factor for Si-related increases in yield in the context of general agricultural production.

### 2.5. Carbohydrates and Soluble Proteins

**Figure 7:**
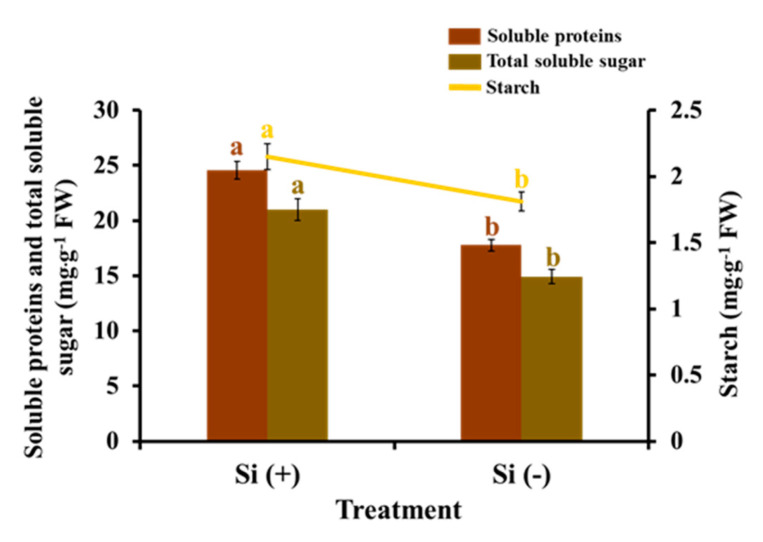
Effect of 30 days of silicon application on the accumulation of carbohydrates and soluble proteins in the coffee plants grown in plant growth chambers. Si (+) and Si (−) refer to 75 and 0 mg·L^−1^, respectively. Vertical bars indicate the means ± standard error (*n* = 6). Different lowercase letters indicate the significant separation within treatments by the Duncan’s multiple range test at *p* ≤ 0.05.

The results relating to the accumulation of carbohydrates and soluble proteins show the strong effect of Si application (Figure 7). On average, soluble sugar, starch, and soluble proteins increased by 40.5%, 18.8%, and 38.2%, respectively, in the Si-treated leaves when compared with non-Si-treated samples. These significant improvements were caused by the comprehensive and positive influence of Si on leaf angle adjustment, the development of vegetative organs, and photosynthetic efficiency.

### 2.6. Root Activity

**Figure 8:**
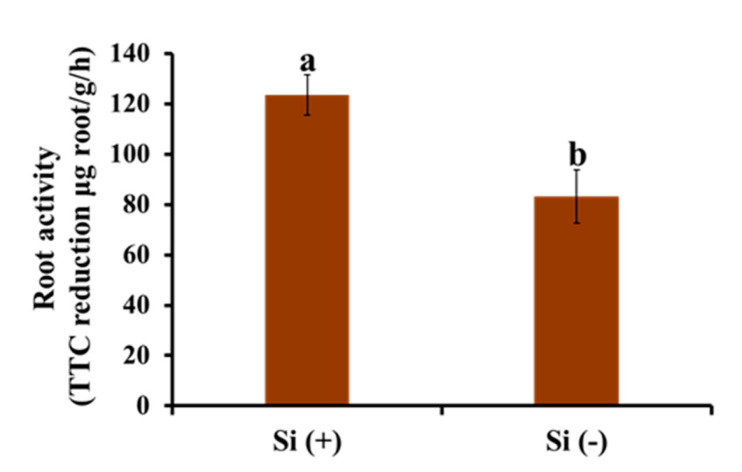
Effect of 30 days of silicon application on root activity in the coffee plants grown in plant growth chambers. Si (+) and Si (−) refer to 75 and 0 mg·L^−1^, respectively. Vertical bars indicate the means ± standard error (*n* = 6). Different lowercase letters indicate the significant separation within treatments by the Duncan’s multiple range test at *p* ≤ 0.05.

It can be seen from Figure 8 that with the application of Si, the root activity of coffee seedlings was significantly enhanced, indicating that Si can affect the root function of plants and promote the utilization of nutrients by roots. This is also consistent with the result that Si promotes perfect root structure development.

### 2.7. Enzymatic Activity

**Figure 9:**
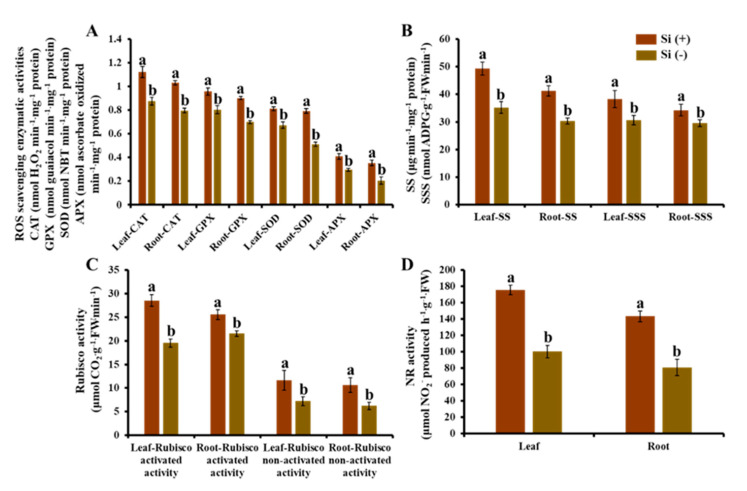
Effect of 30 days of silicon application on enzymatic activities in the coffee plants grown in plant growth chambers. The ROS scavenging enzymatic activities (**A**): Catalase (CAT), guaiacol peroxidase (GPX), superoxide peroxidase (SOD), and ascorbate peroxidase (APX). Carbohydrate synthesis related enzymatic activities (**B**): Sucrose synthase (SS), soluble starch synthase (SSS). Photosynthesis-related enzymatic activities (**C**): activated and non-activated activity of Rubisco. Nitrate reductase (NR) activity (**D**). Si (+) and Si (−) refer to 75 and 0 mg·L^−1^, respectively. Vertical bars indicate the means ± standard error (*n* = 6). Different lowercase letters indicate the significant separation within treatments by the Duncan’s multiple range test at *p* ≤ 0.05.

In our study, concerning the comparison between Si (+) and Si (−), significant differences of some key enzymes related to plant physiology were shown in both leaf and root samples. From Figure 9A, improved ROS scavenging enzymatic activities (CAT, GPX, SOD, and APX) were shown in both Si-treated leaves and roots. The results show that the antioxidant system of Si (+) seedlings increased the number of antioxidant enzymes to balance the damage of peroxidation to plant cells. Moreover, the exogenous Si treatment also significantly raised the enzymatic activities related to carbohydrate synthesis (SS and SSS) and photosynthesis (Rubisco) (Figure 9B,C). It was strongly proved that the application of Si significantly improved seedling health and vitality. At the same time, it was also the basis for the greater biomass and higher photosynthetic efficiency in Si-treated plants. Figure 9D shows that Si has a significant impact on the nitrate reductase (NR) activity of dwarf coffee seedlings, and the NR activity of leaves and roots increased with the application of Si, indicating that Si can improve the nitrogen utilization of seedlings in nutrient solution.

### 2.8. Tissue Mineral Content

Mineral analyses were conducted for both the shoots and roots of the coffee plants (Table 3). Significantly elevated Si occurred in both shoot and root samples after Si supplementation, and the content of Si was always higher in the root than the shoot, indicating that Si in nutrient solution had been absorbed by the plants and accumulated in the plants. In addition, Si also had an influence on the absorption, transportation, and accumulation of other macronutrients. The content of N, P, Ca, and Mg in both the shoots and roots in the Si (+) plants were relatively higher than those in the Si (−) ones. However, Si reduced the K relative content, possibly due to the decreased transpiration rate inducing the dilution of K and competition between Si and K in the Na^+^/K^+^ pump. The accumulation of S did not significantly respond to Si application. For some micronutrients, the lowered content of Fe, Mn, Cu, and B were obtained after Si application in both the shoots and roots of dwarf coffee samples, while there was no obvious change in Zn between Si (+) and Si (−). Moreover, the same pattern of these elements was observed in both shoot and root samples when they responded to Si supplementation, while the relative contents of them in shoots or roots were influenced by the rate of absorption and transportation as well as the dilution effect.

### 2.9. Gene Expression

**Figure 10:**
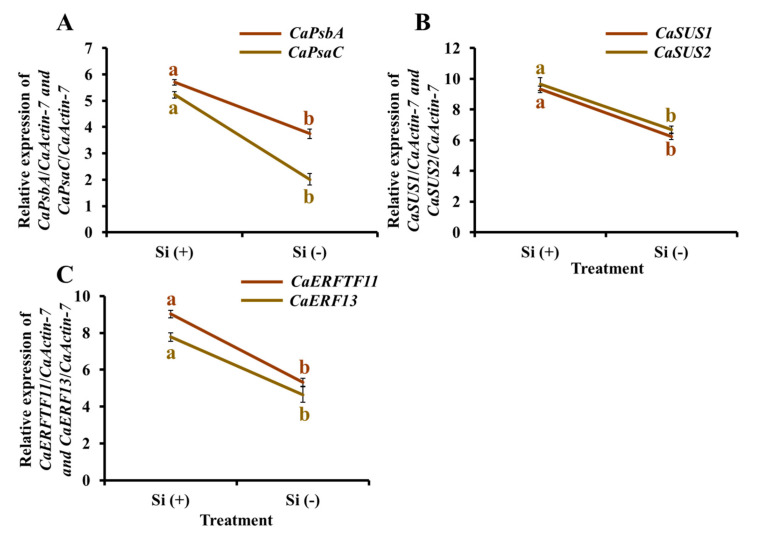
Effect of 30 days of silicon application on gene expression levels in the coffee plants grown in plant growth chambers. Photosynthesis-related genes (**A**): *CaPsbA* and *CaPsaC*. Sucrose synthesis-related genes (**B**): *CaSUS1* and *CaSUS2*. Defense genes (**C**): *CaERFTF11* and *CaERF13*. Si (+) and Si (−) refer to 75 and 0 mg·L^−1^, respectively. Vertical bars indicate the means ± standard error (*n* = 6). Different lowercase letters indicate the significant separation within treatments by the Duncan’s multiple range test at *p* ≤ 0.05.

To validate the differential expression of specific genes related to photosynthetic pathways, sucrose synthetic pathways, and biotic resistance, quantitative (q) RTPCR analyses were performed for key genes of interest (Figure 10). Analysis of the Si-treated samples showed the expected increase in expression of photosynthetic (*CaPsbA* and *CaPsaC*) and sucrose synthetic genes (*CaSUS1* and *CaSUS2*) (Figure 10A,B), which is consistent with the upregulation of Si in photosynthetic activity and carbohydrate accumulation, indicating that the exogenous Si treatment positively adjusted the expression of relative genes. *ERF* genes display enhanced stress tolerance against different pathogens, which are possibly associated with ethylene’s role in defense responses against biotic stresses. Moreover, many ERFs have been shown to be induced upon pathogen attack. Two transcriptional activator PTI5-like defense genes (*CaERFTF11* and *CaERF13*) were selected as research objects (Figure 10C). With a similar expression pattern, the Si (+) samples showed the upregulated expression of these two genes, indicating that Si plays an important role in the prevention and mitigation of plant diseases and insects at the level of genes, further explaining the appearance of pests and diseases only in the Si (−) plants.

## 3. Discussion

In natural conditions, plants suffer from various types of stresses caused by living organisms such as bacteria, viruses, fungi, parasites, and significant and non-significant insects. On invasion by pathogens and herbivorous pests, plants make use of pre-existing physical, chemical, and mechanical barriers to protect themselves. The defense functions of plants are also activated upon attack by pests; plant protections act as a unit to decrease in negative responses to biotic stress [23]. Numerous experiments show the impact of Si on plant tolerance to environmental stresses [24,25,26,27,28,29]. The amount of research articles currently published on the mitigation of plant stress by Si demonstrate the level of interest in this area [24,30,31,32,33]. However, the underlying Si-mediated mode of action to enhance plant resistance to disease and insect pests is unclear. Investigations attribute its influence on the reduction of disease and insect pest attacks via the development of physical barriers by Si deposition and chemical defense through enzyme production. Moreover, researchers have uncovered Si-mediated biochemical and molecular mechanisms in plants that enhance disease tolerance and improve the plant’s ability to survive against insect pest attacks (Figure 2) [22].

### 3.1. Si Mitigates Biotic Stress in Plants: Physical and Mechanical Level

Supplementing plants with Si has been shown to enhance plant tolerance to mammalian, arthropod, and molluscan herbivores, fungal and bacterial pathogens, as well as viruses and nematodes [27,31,34,35,36,37]. Si is translocated from the soil solution as monosilicic acid into plants. Monosilicic acid polymerizes to form phytoliths, which are accumulated within the plant in an irreversible manner [38]. The physical defense induced by Si deposition in plant parts in the form of phytoliths (largely composed of SiO_2_) was one of the first theories proposed for studying stress tolerance to pests [39,40]. The deposition of phytoliths enhances plant immunity and physical resilience and serves as a physical barrier to fungal penetration [41,42]. Si deposition may also wear away the feeding mouthparts or mandibles of insects [43], decrease the plant’s digestibility for both insect and mammalian herbivores [35,44,45], and have an adverse effect on herbivores [46]. Consisting with our study, external Si treatment resulted in strong, thick, and well-developed structures leaves, stems, and roots, which could help mitigate biotic stress in dwarf coffee trees in physical and mechanical ways.

### 3.2. Si Mitigates Biotic Stress in Plants: Physiological Level

A common mechanism by which Si is proposed to function and mitigate biotic stress is ROS and enhanced antioxidant metabolism [47]. The generation of ROS and increasing oxidative metabolism help to reduce oxidative damage to plants [48,49]. ROS generation and increased antioxidant metabolism have been linked to the stress induced by pathogen (bacterial and fungal) infection, as well as plant damage from chewing and sucking insects [48,49,50]. ROS may have a negative and direct effect on biotic stress [51]. In support for our study, the application of Si could enhance the activity of CAT, SOD, APX, and GPX, which play a significant role in biotic stress. GPX is involved in cell-wall reinforcement, in the final stages of lignin biosynthesis, and in the cross-linking of cell wall proteins [52].

Si positively influences root activity and promotes the extensibility of cell walls in the plant root elongation zone to improve root growth [53,54]. Moreover, Si increases xylem conduit hydrophilicity on the surface of cell walls, thus promoting the moisture transmission of plant roots, further enhancing the absorption of N, P, K, Ca, Mg, S, B, Cu, Fe, Mn, and Zn [55]. This was also shown in our results, in which Si significantly increased root volume and biomass and induced mineral absorption. Improved NR activity in both leaves and roots was observed in the Si (+) group, indicating that Si can promote the utilization of nitrogen in nutrient solution by dwarf coffee seedlings. In addition, the application of exogenous Si effectively improved the photosynthesis of plant seedlings under biotic and abiotic stress, showing that indices such as *P*n, *G*s, and *C*i significantly increased while *T*r decreased, which results in benefits of water conservation and improvement of the metabolic process [56]. Consistent with our results, the positive effect of Si on photosynthesis is due to either its protective role in relation to chloroplasts or to the increased concentration of pigments, which is associated with more intense light absorption, or both [57]. Moreover, the enhanced chlorophyll fluorescence characteristics in the Si (+) group can also be considered as strong evidence of promoted photosynthesis [58]. Furthermore, Si increased the content of osmotic adjustment substances (soluble sugar and proteins) by up-regulating corresponding enzymatic activities (e.g., SS and SSS). All of the above regulations revealed that the application of exogenous Si effectively improved the growth of plant seedlings under biotic stress, which is a good foundation for disease and insect defense [56,58].

In addition to the above improvements, current research has looked into the interactions between Si and plant defense signaling transduction pathways, specifically the main plant hormone signaling pathways. Plant hormone signaling has been shown to be important for Si-mediated plant tolerance to disease stress [59,60]. Plants develop a complex and unique blend of salicylic acid (SA) (generally linked with pathogens of (hemi) biotrophic), jasmonic acid (JA) (generally linked with pathogens of necrotrophic and insect herbivores), and ethylene (ETH) (which is usually regarded as ‘fine-tuning’ the JA defense action) in response to attack or infection [34,61]. These findings indicated that Si plays a significant role in multiple phytohormone signaling pathways to mitigate plant biotic stress.

### 3.3. Si Mitigates Biotic Stress in Plants: Molecular Level

Defense-related enzymatic responses induced by Si can be associated with the expression of genes related to enzyme synthesis [62]. In our experiment, the promoted photosynthesis and increased carbohydrate content was due to enhanced relevant enzymatic activities which may be up-regulated by the expression of corresponding genes [63]. Sufficient organic matter and good health in plants are conducive to resistance to pests and diseases. In addition, Si is associated with the metabolic mechanisms of plant-pathogen interactions, triggering host plant defense genes via a sequence of physiological and biochemical reactions and signal transductions, as well as inducing disease resistance in plants [25]. To take an example, *ERF* genes display enhanced stress tolerance against different pathogens, and this is possibly associated with ethylene’s role in defense responses against biotic stresses, which is consistent with our results. Moreover, many ERFs have been shown to be induced upon pathogen attack [64]. Furthermore, numerous studies report that Si could play a role in the primary response, modulating the behavior of the post-elicitation intracellular signaling pathways that control the expression of defense genes involved in cell wall structural modifications, hypersensitivity responses, the synthesis of hormones, and antimicrobial compound synthesis [25]. The expression of genes encoding a novel proline-rich protein (*PRP1*) was increased under the induction of system acquired resistance in Cucumis sativus mediated by Si, which led to cell-wall reinforcement at the site of the penetration of fungi into epidermal cells [65]. Arabidopsis infected with the fungus *Erysiphe cichoracearum* showed changes in the expression of about 4000 genes. The number and/or expression level of defense-related genes enhanced in Si treated plants [66]. Thus, to explore the mechanism of protection of Si in various pathological systems and in insect pest resistance, transcriptomic and proteomic experiments need to be carried out, and this should be the focus of our future research.

### 3.4. An Environmentally Friendly Alternative: Si

The Si has been demonstrated to improve the resilience of crops to a number of biotic stressors and may be used instead of adaptive techniques in certain situations [24,31,32]. Si may readily infiltrate plant cells and influence plant growth by influencing their metabolism through a variety of interactions, triggering the potential to battle stress. Thus, Si has the potential to be used as a fertilizer alone for specific crops and can be used to deliver herbicides and fertilizers in plants. In agriculture, Si may potentially contribute to global food security and safety by assisting with the creation of enhanced crop types with optimal production. Si can provide environmentally beneficial alternatives to many synthetic fertilizers without polluting the environment.

## 4. Materials and Methods

### 4.1. Plant Materials and Growth Conditions

**Figure 11:**
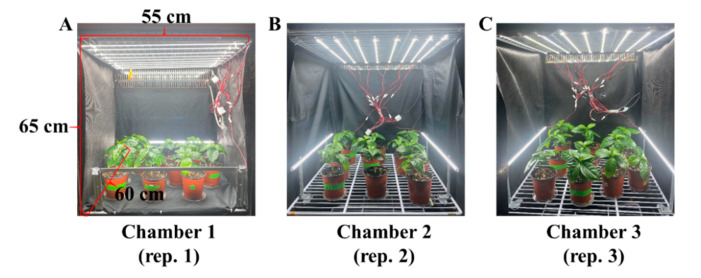
The experimental layout and growth condition of plant growth chambers for coffee plants. (**A**–**C**) refer three plant growth chambers and three replications of plant growth chambers in this experiment.

The seedlings of dwarf coffee (*Punica arabica* ‘Pacas’) with 8 ± 1 leaves per plant were used as experimental materials. They were obtained using seeds harvested from a dwarf mother plant grown in a greenhouse in 2021. The seedlings were stuck in a commercial medium (BVB Medium, Bas Van Buuren Substrates, EN-12580, De Lier, The Netherlands) in 10 cm plastic pots and subsequently acclimated for seven days on a greenhouse bench with an average light intensity of 300 μmol∙m^−2^∙s^−1^ photosynthetic photon flux density (PPFD) of sunlight and a natural photoperiod. In general, plant diseases and insects are naturally transmitted by the wind or insects. The seedlings were grown in the greenhouse along with many plants and infected naturally, and they were then used for the subsequent experiments. To avoid differences caused by original materials and the location inside chambers, a total 30 seedlings were randomly divided into three groups (each group contained two treatments) and transferred into three separate plant growth chambers (C1200H3, FC Poibe Co., Ltd., Seoul, Korea) (with a 25 °C temperature and 80% relative humidity) as three repetitions. Plants of the two treatments were well mixed and randomly laid out in each plant growth chamber. Each chamber was designed as one compartment by plates (Figure 11). To maintain the consistency of lighting conditions, all light-reflecting portions inside the chambers, as well as the plates of each layer, were enclosed in an opaque black curtain. Tailor-made LED lamps (SungKwang LED Co., Ltd., Incheon, Korea) were located in both top and side directions to provide plenty of light with a wide spectrum ranging from 400 to 720 nm and a distinct peak at 435 nm (blue) from 08:00 to 20:00 by adjusting the timer. The light intensity was about 550–600 μmol∙m^−2^∙s^−1^ PPFD which was measured by a quantum radiation probe (FLA 623 PS, ALMEMO, Holzkirchen, Germany) at the top-leaf level of the plant.

### 4.2. Supplementary Si Treatments

The coffee seedlings were drenched with a multipurpose nutrient solution containing either 0 (control) or 75 mg·L^−1^ of Si (Si treatment) supplied from potassium silicate (K_2_SiO_3_) every day at 09:00 a.m. from 22 December 2021 to 21 January 2022 (until the first occurrence of diseases or insects). In detail, the composition of the nutrient solution for the control (the control solution) was as follows (in mg∙L^−1^): 708.0 Ca(NO_3_)_2_∙4H_2_O, 246.0 MgSO_4_∙7H_2_O, 505.0 KNO_3_, 230.0 NH_4_H_2_PO_4_, 1.24 H_3_BO_3_, 0.12 CuSO_4_∙5H_2_O, 4.00 Fe-ethylene diamine tetraacetic acid, 2.20 MnSO_4_∙4H_2_O, 0.08 H_2_MoO_4_, and 1.15 ZnSO_4_∙7H_2_O. The pH of the final solutions (both the control and Si treatment) was adjusted to 6.0 using 1 mol·L^−1^ hydrochloric acid. Additionally, our study was not only designed as a completely randomized layout, but it also had 15 biological replications with consistent growth to minimize external influences.

### 4.3. Measurements of the Morphological Parameters

Morphological parameters were collected after 30 days of growth when the first occurrence of diseases and insects was observed. The plant growth parameters which were shown in Table 1 were measured directly, except for the dry weights of shoots and roots. After drying for one week at 65 °C in a dry oven, the dry weights of the shoots and roots were measured. In addition, the plants were harvested, immediately immersed in liquid nitrogen, and kept in a −80 °C refrigerator for subsequent physiological investigations.

### 4.4. Anatomical Features of Leaves, Stems, and Roots

Six leaf segments (2 cm^2^) without midribs were collected from fully expanded leaves in each treatment for leaf cross-section anatomical observation. These segments were fixed for three days at 4 °C in a formaldehyde solution containing 5% (*v*/*v*) formalin, 5% (*v*/*v*) acetic acid, and 90% (*v*/*v*) ethanol. The leaf samples were sliced to an appropriate thickness by using the freehand slice method after being dehydrated in a graded series of ethanol solutions [95, 75, 50, 25, and 10% (*v*/*v*) ethanol] for each treatment for 40 min and three times, respectively. The slices were mounted on glass slides and observed without staining using an optical microscope (ECLIPSE Ci-L, Nikon Corporation, Tokyo, Japan). ImageJ (ImageJ bundled with 64-bit Java 1.8.0_172, National Institutes of Health, Bethesda, MD, USA) was used to estimate the thickness of the leaves, palisade, and spongy tissues.

For the observation of anatomical features of roots and stems, fresh sections were fixed in formalin-acetic acid-alcohol (FAA) solution containing 50% (*v*/*v*) ethanol, 45% (*v*/*v*) paraformaldehyde, and 5% (*v*/*v*) glacial acetic acid at 4 °C for 24 h. The fixed samples were dehydrated in a graduated solution series, that is, 50% (*v*/*v*) ethanol; 50% (*v*/*v*) ethanol and 10% (*v*/*v*) TBA (tert-butyl alcohol); 50% (*v*/*v*) ethanol and 20% (*v*/*v*) TBA; 50% (*v*/*v*) ethanol and 35% (*v*/*v*) TBA; 50% (*v*/*v*) ethanol and 50% (*v*/*v*) TBA; 25% (*v*/*v*) ethanol and 75% (*v*/*v*) TBA; 25% (*v*/*v*) ethanol and 75% (*v*/*v*) TBA; and 0.1% (*w/v*) safranin O, each for 45 min and cleared with 100% (*v*/*v*) TBA for another 45 min; the procedure was repeated twice. The samples were then infiltrated with 67% (*v*/*v*) TBA + 33% (*w/v*) paraffin (Paraplast X-tra, Kendall, FL, USA) overnight and 100% (*w/v*) of paraffin for 24 h (renewed every 2 h during the daytime) at 60 °C and then embedded in paraffin. Paraffin sections with a thickness of 10 mm were cut using the rotary microtome (RM2125RT, Leica, Nussloch, Germany), floated on a 42 °C water-bath for relaxing compression and mounting on the Superfrost Plus microscope slide (Menzel-Gläser, Braunschweig, Germany). Dried sections were de-paraffinized and re-hydrated through a graded series [67]. Moreover, after the aforesaid treatment, stem sections were further stained with 1% (*w/v*) safranin O in 50% (*v*/*v*) ethanol for 3 h and 0.5% (*w/v*) methyl green for 45 min [68]. After washing and dehydration, the stained sections were permanently mounted with a coverslip using the Canada balsam. Finally, specimens (each section of 10 mm) were observed on an optical microscope (ECLIPSE Ci-L, Nikon Corporation, Tokyo, Japan). The anatomical structures of stems and roots were observed using ImageJ (ImageJ bundled with 64-bit Java 1.8.0_172, National Institutes of Health, Bethesda, MD, USA).

### 4.5. Stomatal Density and Morphological Characteristics

Six plants were randomly selected from each treatment, with the lower epidermis of leaves without midribs from fully expanded leaves in a similar position to observe the stomatal morphology. The leaf samples were fixed for two days at 4 °C in a mixed solution containing 45% (*v*/*v*) acetone, 45% (*v*/*v*) ethanol, and 10% (*v*/*v*) distilled water for decoloring. The excised samples were observed at different magnifications using an optical microscope (ECLIPSE Ci-L, Nikon Corporation, Tokyo, Japan) and were analyzed with ImageJ (ImageJ bundled with 64-bit Java 1.8.0_172, National Institutes of Health, Bethesda, MD, USA). By dividing the number of stomata by the area where the number of stomata was recorded, the stomatal density was determined. The morphological parameters of stoma include the length and width of guard cell pairs and stomatal pores measured by Sack and Buckley’s description [69].

### 4.6. Chlorophyll Content

Exactly 0.1 g of fresh leaves were collected for measuring the chlorophyll content (mg∙g^−1^ fresh weight), and there were six replicates in each treatment. All samples were dipped in 10 mL of *N*,*N*-dimethyl formamide solution and left in the dark for 48 h at 4 °C before being tested for Chl a and Chl b. Using a UV spectrophotometer (Libra S22, Biochrom Ltd., Cambridge, UK), the absorbance of the upper layer solution at 645 and 663 nm were recorded, respectively. The chlorophyll content was estimated according to Sim et al. [70].

### 4.7. Measurements of Photosynthesis and Chlorophyll Fluorescence

Photosynthetic parameters (including the net photosynthetic rate (*P*n), transpiration rate (*T*r), stomatal conductance (*G*s), and intercellular CO_2_ concentration (*C*i)) of fully expanded leaves of the dwarf coffee tree were measured by a leaf porometer (SC-1, Decagon Device Inc., Pullman, WA, USA). These parameters were measured in the plant growth chambers to maintain the same and steady conditions from 9:30 to 11:30 a.m. Moreover, chlorophyll fluorescence characteristics were detected by the miniaturized pulse-amplitude-modulated photosynthesis yield. The measurements involved the maximal PSII quantum yield (*F*v/*F*m), photochemical efficiency of PSII (*F*v′/*F*m′), non-photochemical quenching (NPQ), and coefficient of photochemical quenching (*qP*) being monitored by a photo system (Fluor Pen FP 100, Photon Systems Instruments, PSI, Drásov, Czech Republic) after 30 min dark adaption. All the parameters were calculated using the methods reported by Maxwell et al. [71].

### 4.8. Accumulation of Carbohydrates and Soluble Proteins

For starch and soluble sugars measurements, leaves at the same developmental stage were obtained at the end of the day or night, and tested using the anthrone colorimetric method according to Vasseur and Ren et al. [72,73]. The method of extracting soluble proteins was as followed: Fresh leaves were collected, immediately immersed in liquid nitrogen, and ground into a fine powder over an ice bath. Then, 100 mg of the powder was homogenized in 50 mM of PBS (1 mM EDTA, 1 mM polyvinylpyrrolidone, and 0.05% (*v*/*v*) triton-X, pH = 7.0). The resulting mixture was then centrifuged (13,000 rpm, 4 °C, 20 min) to obtain the supernatant that would be used afterwards for total protein estimation and enzyme activity assay [74]. The total protein estimations were conducted using Bradford’s reagent [75,76]. The content of carbohydrates and soluble proteins was measured using a UV spectrophotometer (Libra S22, Biochrom Ltd., Cambridge, UK).

### 4.9. Root Activity

Root activity was analyzed using the triphenyl tetrazolium chloride (TTC) method [77]. TTC is a chemical that is reduced by dehydrogenases, mainly succinate dehydrogenase, when added to a tissue. Dehydrogenase activity is regarded as an index of root activity. In brief, 0.5 g fresh root was immersed in 10 mL of equally mixed solution of 0.4% TTC and phosphate buffer and kept in the dark at 37 °C for 2 h. Subsequently, 2 mL of 1 mol/L H_2_SO_4_ was added to stop the reaction with the root. The root was dried with filter paper and then extracted with ethyl acetate. The red extractant was transferred into the volumetric flask to reach 10 mL by adding ethyl acetate. The absorbance of the extract at 485 nm was recorded. Root activity was expressed as the TTC reduction intensity. Root activity = amount of TTC reduction (µg)/fresh root weight (g) × time (h).

### 4.10. Enzyme Activities

Catalase (CAT) activity was measured in the homogenates by directly detecting the induction of H_2_O_2_ in A_240nm_, as described by Aeibi et al. [78]. The reaction medium contained 50 mM potassium phosphate buffer (PH 7.0), 10 mM H_2_O_2,_ and 0.1 mL enzyme extract in a final volume of 3 mL at 25 °C. The extinction coefficient (40 mM^−1^ cm^−1^) for H_2_O_2_ was used to calculate CAT activity. Castillo et al. [79] proposed a method for measuring guaiacol peroxidase activity (GPX). The 3 mL reaction mixture contained 10 mM guaiacol, 50 mM H_2_O_2_, 50 mM phosphate buffer (PH 6.0), and 50 μL enzyme extract. The extinction coefficient of 26.6 mM^−1^ cm^−1^ was used to compute the absorbance at A_470nm_. The capacity of superoxide dismutase (SOD) to block the photochemical reduction of nitroblue tetrazolium (NBT) was measured, as described by Becana et al. [80]. The 3 mL reaction mixture solution contained 50 mM potassium phosphate buffer (PH 7.8), 50 mM methionine, 75 μM NBT, 20 μM riboflavin, 0.1 mM EDTA, and 0.1 mL of enzyme extract. The reaction mixture was performed for 15 min. Blanks and controls were run in the same fashion as the other groups but without any light and enzyme extract, respectively. By monitoring at A560 nm, one unit of SOD was defined as the quantity of enzyme that provided a 50% inhibition of NBT reduction. The activity of ascorbate peroxidase (APX) was measured for 1 min at A_290nm_ (extinction coefficient 2.9 mM^−1^ cm^−1^) according to Nakano [81]. The reaction mixture contained 50 mM sodium phosphate buffer (PH 7.0), 0.1 mM EDTA, 1 mM ascorbate acid, 2.5 mM H_2_O_2_, and 50 μL of enzyme extract.

The enzymatic activities of the key enzymes related to sucrose synthesis (SS), starch synthesis (SSS), and photosynthesis (Rubisco) were also measured by using a UV spectrophotometer (Libra S22, Biochrom Ltd., Cambridge, UK). The SS was determined in a 1 mL reaction mixture containing 500 μL enzyme extract at 34 °C for 1 h. A 300 μL 30% (*v*/*v*) KOH was added to this mixture, followed by placement in a water bath at 100 °C for 10 min and gradual cooling to room temperature. The mixture was subjected to incubation at 40 °C for 20 min after 200 μL 0.15% (*v*/*v*) anthrone-sulfuric acid solution was applied, and the enhancement of A_620nm_ was monitored. The Rubisco total activity was measured by injecting 100 μL of the supernatant into 400 μL of an assay mixture consisting of 50 mM Tris-HCl (pH 8.0), 5 mM DTT, 10 mM MgCl_2_, 0.1 mM EDTA, and 20 mM NaH_14_CO_3_ (2.0 GBq mmol^−1^) at 30 °C. After a 5 min activation period, the reaction was initiated by adding RuBP to 0.5 mmol L^−1^ and terminating after 30 s with 100 μL of 6 mol L^−1^ HCl. In addition, the activities of soluble starch synthase (SSS) were determined using the Doehlert et al. and Liang et al. [82,83] protocols. The activities of nitrate reductase (NR) in plants were assayed in vitro in accordance with Hogberg et al. [84]. NR activity was expressed as the amount of nitrite formed per gram of dry weight per hour.

### 4.11. Determination of Tissue Mineral Content

An analysis of tissue mineral content was performed after 30 days of cultivation with or without Si application. To determine the content of Si, macronutrients (N, P, K, Ca, Mg, and S), and micronutrients (Fe, Mn, Zn, Cu, and B), dried shoot and root samples from each treatment were ground separately and respectively. The samples were ashed at 525 °C for 4 h in a Nabertherm muffle furnace (Model LV 5/11/B180, Lilienthal, Bremen, Germany). The ash was dissolved in 5 mL 25% HCl, followed by dilution with 20 mL of warm distilled water. The content was then measured six times for each treatment using an inductively coupled plasma (ICP) spectrometer (Optima 4300DV/5300DV, Perkin Elmer, Germany) [85].

### 4.12. Real-Time Quantitative PCR Verification

The expression of key genes related to sucrose synthesis (*CaSUS1* and *CaSUS2)* [63], photosynthesis (*CaPsbA* and *CaPsaC*) [86], and transcriptional activator PTI5-like defense genes (*CaERFTF11* and *CaERF13*) [64] were searched. The primers were designed through https://www.ncbi.nlm.nih.gov/tools/primer-blast/ (accessed on 3 February 2022) according to the sequences which were obtained from https://www.ncbi.nlm.nih.gov/ (accessed on 3 February 2022). Leaf, stem, and root mixed samples were collected from the coffee seedlings, which were from the control group or experimental group, respectively. All samples were immediately frozen in liquid nitrogen. The total RNA was extracted using an Easy-Spin total RNA extraction kit (iNtRON Biotechnology, Seoul, Korea), then used for first-stand cDNA synthesis with the GoScript Reverse Transcription System (Promega, Madison, WI, USA) according to the manufacturer’s protocols. Real-time quantitative PCR was conducted in a real-time PCR system (CFX96, Bio-Rad, Hercules, CA, USA). Reaction volumes (20 μL) contained 1 μL of cDNA, 1 μL of each amplification primer (10 μM), 10 μL of 2 × AMPIGENE qPCR Green Mix Lo-ROX (Enzo Life Sciences Inc., Farmingdale, NY, USA), and 7 μL ddH_2_O (double distilled water). The 2−ΔΔCt method was used for the data analysis, and the *ACTIN* gene (*CaActin-7*) was selected. All the target gene primers are listed in Table 4.

### 4.13. Statistical Analysis

Significant differences among the treatments were assessed by an analysis of variance (ANOVA) followed by the Duncan’s multiple range test at a probability (*p*) ≤ 0.05 with a statistical program (SAS, Statistical Analysis System, V. 9.1, Cary, NC, USA). The differences between the control and the Si treatment were tested by Student’s *t-test* (*p*) ≤ 0.05. The Fisher’s least significant difference test was used for the *F*-test between treatments. Moreover, the experimental assays used to obtain all results were repeated six times and are presented as the mean ± standard error.

## 5. Conclusions

Si is a bioactive element in various biological systems which has a positive impact on plant health by effectively mitigating biotic and abiotic stresses. It has been shown to improve plant growth and development, enhance the expression of natural defense mechanisms, promote plant resistance to herbivorous insects, and prevent the infection by pathogens by improving physiological characteristics and up-regulating some defense genes, and it, therefore, shows promise in its application as a part of an integrated pest management. However, its mode of action in plants remains a matter of speculation, and there remain many details that require our further exploration: (1) Is soluble Si a key factor in promoting the plant production of biochemical defenses against herbivorous insects? (2) Which or how many defense-related genes are up-regulated by Si application after insect invasion (e.g., the role of these genes in JA and SA biosynthesis)? (3) When plants are subjected to insect stress, do other biological stressors bind to Si to induce a greater degree of plant defense response?

## Figures and Tables

**Table 1:** Influence of 30 days silicon application on morphological characteristics in the coffee plants grown in plant growth chambers.

Treatment ^1^	Shoot					Leaf					Root			Symptoms of Disease or Insects
	Plant Height (cm)	Canopy Diameter (cm)	Stem Diameter (mm)	FW (g) ^2^	DW (g) ^3^	Number	Length (cm)	Width (cm)	No. of Withered Leaves (Chlorosis)	The Adaxial Angle of the Top^1st^ Leaves (°)	Length (cm)	FW (g) ^2^	DW (g) ^3^	
MNS-Si (+)	13.8 a ^4^	19.2 a	3.1 a	8.9 a	2.7 a	16.3 a	10.1 a	5.3 a	0.1 b	45.3 b	20.0 a	5.0 a	0.9 a	No
MNS-Si (−)	9.5 b	11.3 b	2.2 b	5.3 b	1.5 b	11.4 b	6.9 b	3.8 b	3.4 a	88.7 a	15.0 b	1.2 b	0.2 b	Yes

^1.^ MNS-Si (+) and MNS-Si (-) refer to the multipurpose nutrient solution (MNS) with 75 and 0 mg·L^−1^, respectively. ^2.^ Fresh weight (g). ^3.^ Dry weight (g).^4.^ Mean separation within columns by the Duncan’s multiple range test at *p* ≤ 0.05. Each treatment was measured six times.

**Table 2:** Influence of 30 days of silicon application on the photosynthetic and chlorophyll fluorescence characteristics of the coffee plants grown in plant growth chambers.

Treatment ^1^	*P*n ^2^ (μmol CO_2_.m^−^^2^·s^−^^1^)	*T*r ^3^ (mmol H_2_O m^−^^2^·s^−^^1^)	*G*s ^4^ (mol.H_2_O m^−^^2^·s^−^^1^)	*C*i ^5^ (μmol CO_2_mol^−^^1^)	*F*v/*F*m ^6^	*F*v′/*F*m′ ^7^	NPQ ^8^	*qP* ^9^
MNS-Si (+)	21.54 a ^10^	1.91 b	1.04 a	536.47 a	0.99 a	0.88 a	2.97 a	0.74 a
MNS-Si (-)	18.67 b	2.68 a	0.82 b	467.26 b	0.79 b	0.71 b	1.92 b	0.54 b

^1^ MNS-Si (+) and MNS-Si (−) refer to the multipurpose nutrient solution (MNS) with 75 and 0 mg·L^−1^, respectively. ^2^ Net photosynthetic rate (*P*n). ^3^ Transpiration rate (*T*r). ^4^ Stomatal conductance (*G*s). ^5^ Intercellular CO_2_ concentration (*C*i). ^6^ The maximal PSII quantum yield (*F*v/*F*m). ^7^ The photochemical efficiency of PSII (*F*v′/*F*m′). ^8^ Non-photochemical quenching (NPQ). ^9^ Coefficient of photochemical quenching (*qP*). ^10^ Mean separation within columns by the Duncan’s multiple range test at *p* ≤ 0.05. Each treatment was measured six times.

**Table 3:** Influence of 30 days of silicon application on mineral content in both shoot and root of coffee plants.

Sample (A) ^1^	Si (mg L^−1^) (B)	Si Content (mg g^−1^ Dry Weight)	Macronutrients (mg g^−1^ Dry Weight)						Micronutrients (μg g^−1^ Dry Weight)				
			N	K	Ca	Mg	P	S	B	Cu	Fe	Mn	Zn
Shoot	0	0.45 d ^2^	219.24 cd	115.35 c	60.41 b	19.14 b	12.00 b	3.59 b	0.19 c	0.21 a	0.67 a	0.66 a	0.08 b
	75	0.90 b	304.19 b	91.29 d	72.50 a	29.36 a	15.17 a	3.65 b	0.10 cd	0.18 ab	0.41 b	0.51 ab	0.07 b
Root	0	0.58 c	228.36 c	209.57 a	40.29 d	9.12 cd	7.56 d	10.61 a	0.63 a	0.10 b	0.69 a	0.49 b	0.13 a
	75	1.89 a	327.24 a	187.87 b	52.54 c	11.19 c	10.14 c	9.99 a	0.50 b	0.09 bc	0.41 b	0.38 c	0.14 a
*F*-test	A	***	***	***	***	***	***	***	***	**	NS	***	***
	B	***	**	***	***	**	***	NS	**	*	***	**	NS
	A × B	***	**	***	***	***	***	***	***	**	**	**	***

^1.^ The factor A in the *F*-test refers to the sample; the factor B means level of Si supplemented to the multipurpose nutrient solution (MNS). ^2.^ Mean separation within columns by the Duncan’s multiple range test at *p* ≤ 0.05. NS, *, **, ***; non-significant or significant at *p* ≤ 0.05, 0.01, 0.001, respectively. Silicon (Si), macronutrients (nitrogen (N), phosphorus (P), potassium (K), calcium (Ca), magnesium (Mg), and sulfur (S)), and micronutrients (iron (Fe), manganese (Mn), zinc (Zn), copper (Cu), and boron (B)). Each treatment was measured six times.

**Table 4:** List of the primers used to quantify expression levels of genes.

Name	Gene ID	Gene bank Number	Description	Forward Primer (5′ to 3′)	Reverse Primer (5′ to 3′)
*CaActin-7*	113701041	-	Transcript variant X1	TAGCAACTGGGATGACATGGA	AGTCAAGAGCCACATAGGCA
*CaPsbA*	4421839	-	Photosystem II protein D1	CTGCAGCTATAGGTTTGCACTT	TGCAACAGCAATCCAAGGTC
*CaPsaC*	4421852	-	Photosystem I subunit VII	TAGAAATGATACCTTGGGACGGA	ACCCATACTGCGGGTTGTT
*CaSUS1*	113691468	AM087674.1	Sucrose synthase	GGGTTGCACTTGCTATTCGTC	AGCATCATTGTCTTGCCCTTG
*CaSUS2*	113718268	AM087675	Sucrose synthase 2-like	GCCGCTCTGAAGACCATTTAG	AGGTGTATCTTGTGGGAGCTTG
*CaERFTF11*	113726097	KF743550.1	Defense genes transcriptional activator PTI5-like. ERF transcription factor 11	TTCGCGATTCGACGAGAAATG	TTCCTGTCGATCTAGGTTCAGC
*CaERF13*	113726097	KF278730.1	Defense genes transcriptional activator PTI5-like. Ethylene response factor 13	GGCCATGGGGAAAATACGCA	CGTCCCAGACGAAACTTCAG

## Data Availability

Data sharing is not applicable to this article.
